# Thirdhand Smoke May Promote Lung Adenocarcinoma Development through HN1

**DOI:** 10.1155/2023/3407313

**Published:** 2023-01-30

**Authors:** Jiamin Peng, Jia Shuopeng, Huiju Wang, Xin Wang, Ligang Wang

**Affiliations:** ^1^Department of Clinical Laboratory, Zhejiang Tongde Hospital, Hangzhou, Zhejiang 310014, China; ^2^Department of Clinical Trials Center, National Cancer Center/National Clinical Research Center for Cancer/Hebei Cancer Hospital, Chinese Academy of Medical Sciences, Langfang 065001, China; ^3^Key Laboratory of Gastroenterology of Zhejiang Province, Zhejiang Provincial People's Hospital, People's Hospital of Hangzhou Medical College, Hangzhou, China; ^4^Department of Clinical Trials Center, National Cancer Center/National Clinical Research Center for Cancer/Cancer Hospital, Chinese Academy of Medical Sciences and Peking Union Medical College, Beijing 100041, China; ^5^Cancer Center, Department of Ultrasound Medicine, Zhejiang Provincial People's Hospital, Affiliated People's Hospital, Hangzhou Medical College, Hangzhou, Zhejiang, China

## Abstract

Thirdhand smoke (THS) refers to residual tobacco smoking pollutants that can be adsorbed to indoor surfaces and dust and persist for years after active smoking. THS-related chemicals such as N-nitrosonornicotine (NNN) and 4-(methylnitrosamino)-1-(3-pyridyl)-1-butanone (NNK) are tobacco-specific lung carcinogens that involved in lung cancer development and progression. In this study, we computed the differentially expressed genes (DEGs) between THS and paired control samples. THS-related overexpressed genes (OEs) were overlapped with OEs of lung adenocarcinoma (LUAD) and lung squamous cell carcinoma (LUSC). Survival analyses of these overlapped genes were performed using LUAD and LUSC data. 6 genes were selected for validation based on their expression levels and prognostic value. Hematological and neurological expressed 1 (HN1) was further selected due to its novelty in LUAD research. The potential roles of HN1 in LUAD were explored in several ways. In summary, HN1 is overexpressed in THS samples and is associated with the prognosis of patients with LUAD. It may promote cancer progression through several pathways and could serve as a potential therapeutic target especially for THS-related LUAD. In-depth mechanistic studies and clinical trials are warranted.

## 1. Introduction

Thirdhand smoke (THS) refers to residual tobacco smoking pollutants that can adsorbed to indoor surfaces, clothes, and dust and persist for years after active smoking [1]. These deposited chemicals such nicotine could reemit into the air and react with nitrous acid to form carcinogenic tobacco-specific nitrosamines (TSNAs) including N-nitrosonornicotine (NNN) and 4-(methylnitrosamino)-1-(3-pyridyl)-1-butanone (NNK), which are tobacco-specific lung carcinogens that are involved in the development of lung cancer [2]. The presence of THS in indoor environment is widespread, and it is a severely underestimated health hazard. Currently, there are no effective ways to eliminate THS.

A randomized clinical trial identified 389 genes that were differentially expressed in human respiratory epithelium in response to acute THS exposure or clean air for 3 hours. Gene ontology analysis indicated that these genes were enriched in cell stress and survival-related signaling pathways such as respiratory electron transport chain, DNA repair, and activation of cell viability [3]. It showed that human respiratory epithelium could respond rapidly to THS. Importantly, Hang et al. reported that THS could induce lung cancer development and increase lung cancer incidence in mice [4, 5]. However, the association between THS and lung cancer is still poorly understood due to limited numbers of studies to date.

In this study, we explored the potential carcinogenesis roles of THS in human lung cancer by investigating overlapped-overexpressed genes in THS and lung cancer. This research will provide a mechanistic link between THS and human lung cancer, which would pave the way for future further investigations.

## 2. Results

### 2.1. THS-Related Genes Are more Associated with Lung Adenocarcinoma (LUAD) Not Lung Squamous Carcinoma (LUSC)

**Figure 1:**
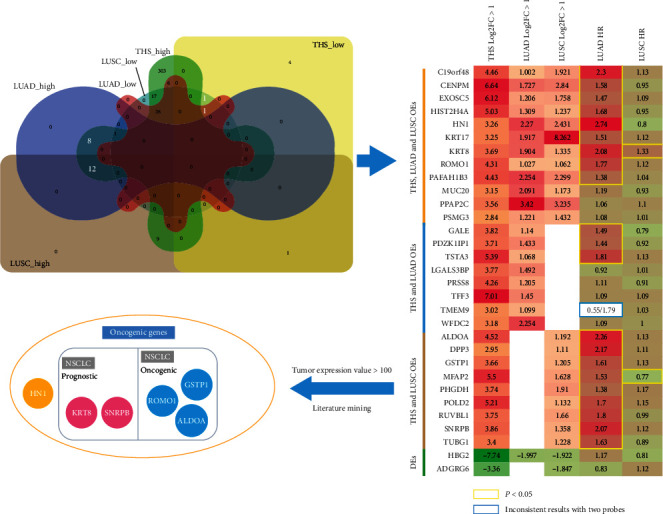
Screening for potential therapeutic targets of THS-related lung cancer.

Of 382 THS-related overexpressed genes (OEs), 12 genes are overexpressed both in LUAD and LUSC, 8 genes are overexpressed in LUAD, and 9 genes are overexpressed in LUSC. Of 7 THS-related downexpressed genes (DEs), one gene is downexpressed both in LUAD and LUSC while another gene is only downexpressed in LUSC (Figure 1, upper left). As shown in the heat map (Figure 1, upper right), 21 genes are associated with poor overall survival (OS) of LUAD. Even 18/21 genes are overexpressed in LUSC and 9/21 genes are exclusively overexpressed in LUSC based on filter of log2FC > 1, and only KRT8 correlated with poor OS of LUSC while MFAP2 associated with better OS. Detailed survival analysis results of 31 common DEGs in THS and lung cancer are shown in Table 1. This is very interesting since THS-related DEGs are mainly related to prognosis of LUAD.

Gene expression levels are very important for their biological functions, the 21 OS-related genes were further filtered based on their expression in LUAD, and 6 genes with expression value > 100 were obtained (middle-left). These 6 genes might play critical roles in THS-associated LUAD. Literature mining confirmed that all these 6 genes are associated with cancer. KRT8 and SNRBP are overexpressed and correlated with poor prognosis of NSCLC; ROMO1, GSTP1, and ALDOA are oncogenic in NSCLC. HN1 can promote cancer progression in other cancer types, but there is no report on its association with NSCLC.

### 2.2. Validation of the Prognostic Value of the 6 Genes in LUAD and Other Cancer Types

**Figure 2:**
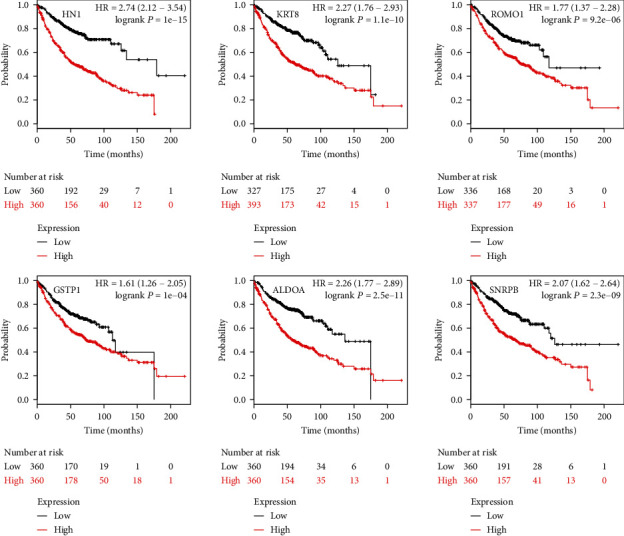
Kaplan-Meier survival analysis of HN1, KRT8, ROMO1, GSTP1, ALDOA, and SNRPB in lung adenocarcinoma. Expression levels of the 6 genes are all associated with poor prognosis of patients with lung adenocarcinoma.

We validated the prognostic value of the 6 genes using a merged LUAD dataset from KmPlot. Survival analyses results confirmed that these 6 genes are all associated with poor prognosis of patients with LUAD (Figure 2, red line indicates high expression; black line indicates low expression). Specifically, HN1 (HR = 2.74, logrank *p* = 1E − 15); KRT8 (HR = 2.27, logrank *p* = 1.1E − 10); ROMO1 (HR = 1.77, logrank *p* = 9.2E − 06); GSTP1 (HR = 1.61, logrank *p* = 1E − 04); ALDOA (HR = 2.26, logrank *p* = 2.5E − 11); SNRPB (HR = 2.07, logrank *p* = 2.3E − 09).

We also explored the expression and prognostic value of these 6 genes in other types of cancer using GEPIA 2.0 webtool. As shown in Supplementary Figure 1, 6 genes were overexpressed in most cancer types, such as pancreatic cancer and colorectal cancer (heat map of log2 transformed fold changes, red box: high expression in cancer tissue against normal control, *p* < 0.05; blue box: low expression in cancer tissue, *p* < 0.05). Supplementary Figure 2 is the hazard ratio heat map of 6 genes in 29 cancer types. The upper panel is overall survival (OS), and the lower panel is recurrent free survival (RFS). Red box indicates association with poor prognosis while blue box indicates association with better prognosis (*p* < 0.05). As we can see in Supplementary Figure 2, these 6 genes are associated with poor prognosis of several cancer types. These results demonstrated that THS-related genes not only important in LUAD but may also play critical roles in many other cancer types.

### 2.3. Expression and Function Analyses of HN1 in LUAD

**Figure 3:**
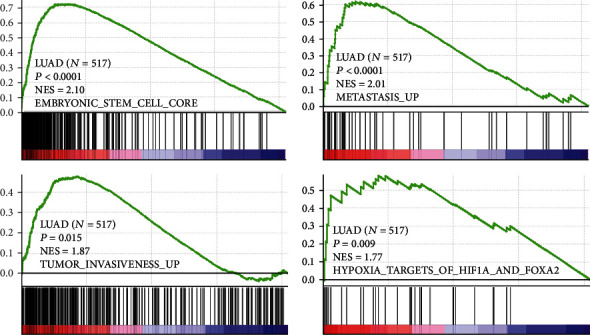
GSEA results indicate that HN1 expression is associated with stem cell, metastasis, invasiveness, and hypoxia-related pathways.

**Figure 4:**
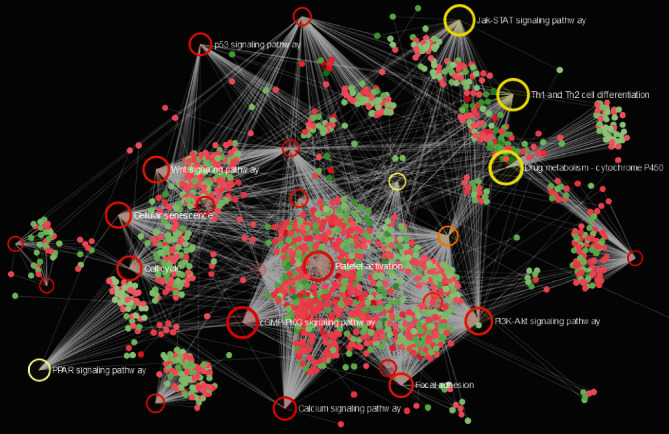
Pathway enrichment visualization of differentially expressed genes between HN1 high/low groups using NetworkAnalyst.

Literature mining showed that HN1 is the only one in the 6 genes that has not been reported its association with lung cancer. Thus, we selected HN1 to explore its potential function and oncogenic roles in LUAD. Further analyses show that HN1 expressions in different stages of LUAD were all significantly higher than in normal control (Supplementary Figure 3), and its high expression was associated with poor prognosis of patients with LUAD (Supplementary Figure 4). GSEA analyses were performed using DEGs between HN1 high and low groups. Result shows that HN1 expression is positively correlated with embryonic stem cell signatures, metastasis, invasiveness, and hypoxia pathways (Figure 3, *p* < 0.0001, *p* < 0.0001, *p* = 0.015, and *p* = 0.009, respectively). Pathway enrichment analysis using clusterProfiler shows that HN1-related DEGs were enriched in cell adhesion molecules (CAMs), Hedgehog signaling pathway, proteoglycans in cancer, cellular senescence, PI3K-Akt signaling pathway, etc. (Supplementary Table 1). Visualization of enrichment pathway network is presented in Figure 4.

**Figure 5:**
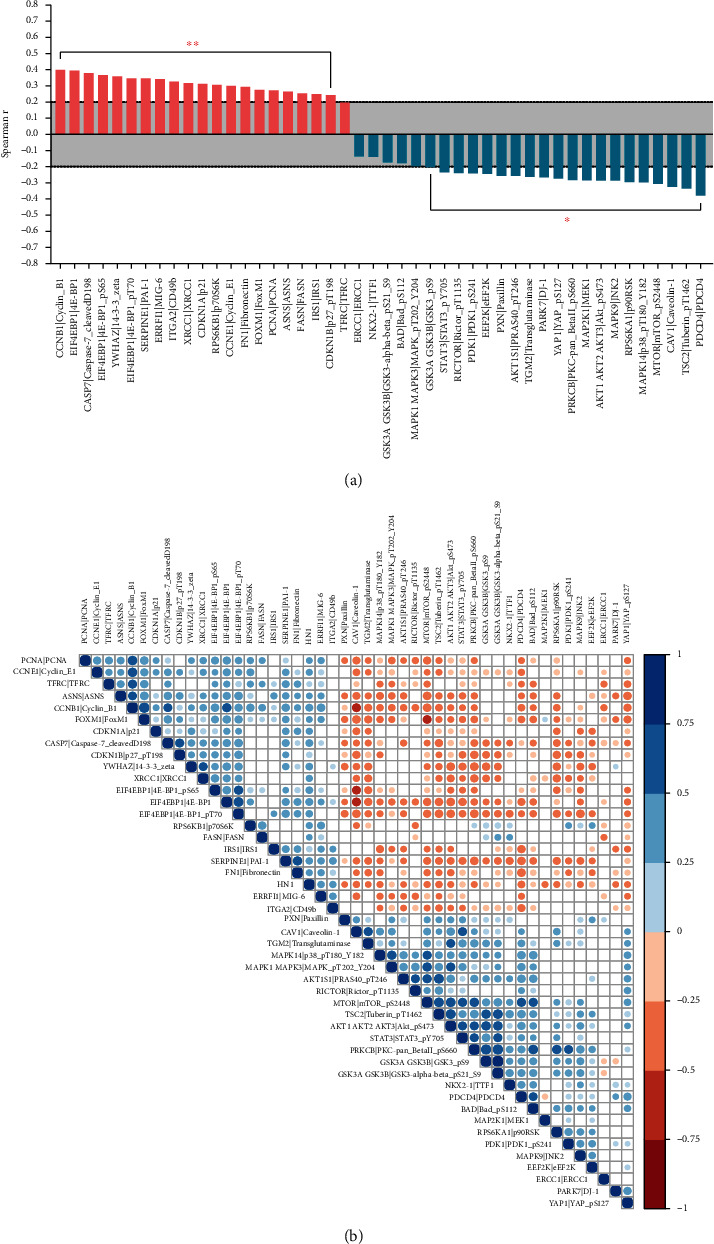
Correlation between HN1 and proteins in key pathways. (a) Box plot of spearman *r* value from correlation analysis between HN1 and proteins in key pathways. (b) Correlation map of HN1 with proteins in key pathways.

Figure 5 shows the correlation between HN1 and RPPA protein abundances.  Figure 5(a) is the box plot of spearman *r* computed by using LUAD-HN1 expression data and RPPA data. HN1 is positively correlated with cell cycle and metastasis-related proteins such as CCNB1, CCNE1, FN1, and FOXM1, while it is negatively correlated with PI3K-AKT pathway-related proteins such as AKT pS473 and PRAS40 pT246.  Figure 5(b) is the correlation map of HN1 and RPPA proteins (blue represents positive correlation while red represents negative correlation, correlations with *p* value < 0.05 were presented in the map). These protein level analysis results are in accordance with the above pathway enrichment analyses.

### 2.4. Association Analyses of HN1 and Cancer Immunotherapy-Related Factors

**Figure 6:**
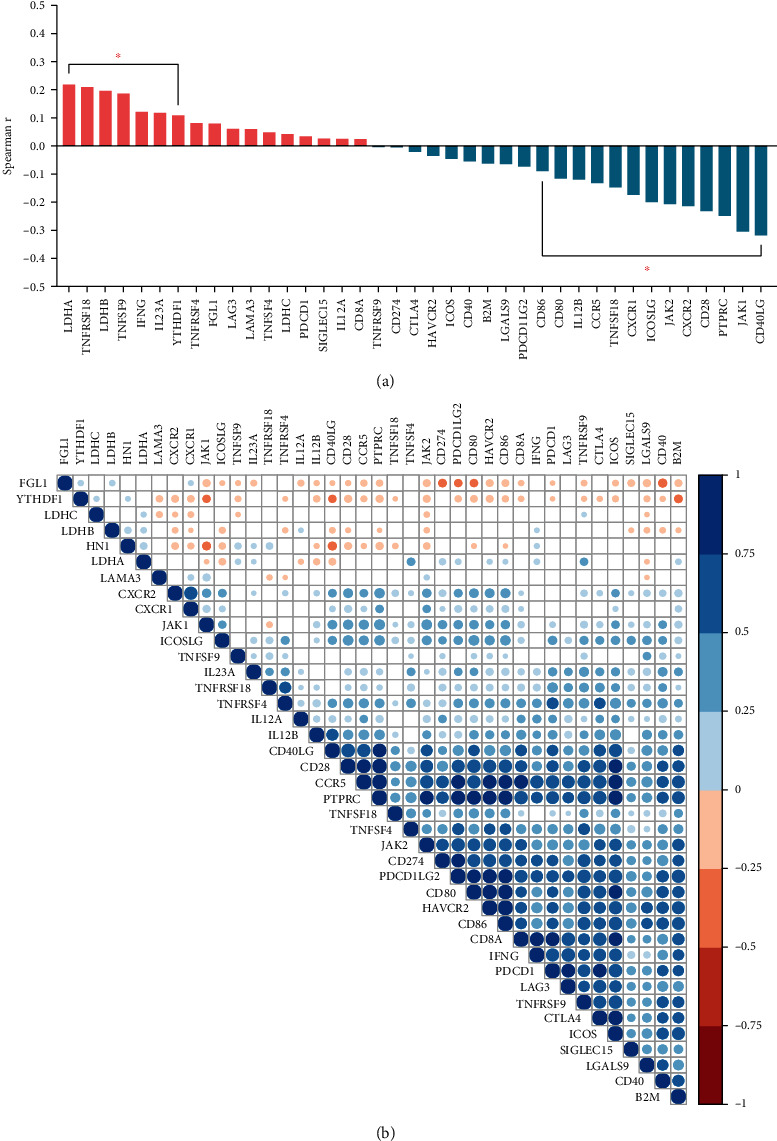
Correlation between HN1 and ICB efficacy-related genes. (a) Box plot of spearman *r* value from correlation analysis between HN1 and ICB efficacy-related genes. (b) Correlation map of HN1 with ICB efficacy-related genes.

The possible association between HN1 and cancer immunotherapy-related factors such as immune checkpoint blockade (ICB) therapy efficacy-related genes and abundance of cancer microenvironment cells were also investigated.  Figure 6 shows the correlation between HN1 and ICB efficacy-related genes (Figure 6(a) is box plot of spearman *r* value; Figure 6(b) is correlation map). Figure 6(a) shows that HN1 expression is positively correlated with LDHA, LDHB, TNFSF9, TNFRSF18, IFNG, etc., while negatively correlated with JAK1, JAK2, CD40LG, etc., indicating the possibility that HN1 might serve as a prognostic biomarker or potential therapeutic target for cancer immunotherapy. Figure 6(b) is the correlation map of HN1 and ICB efficacy-related molecules. It shows the correlation clusters of these molecules in LUAD.

**Figure 7:**
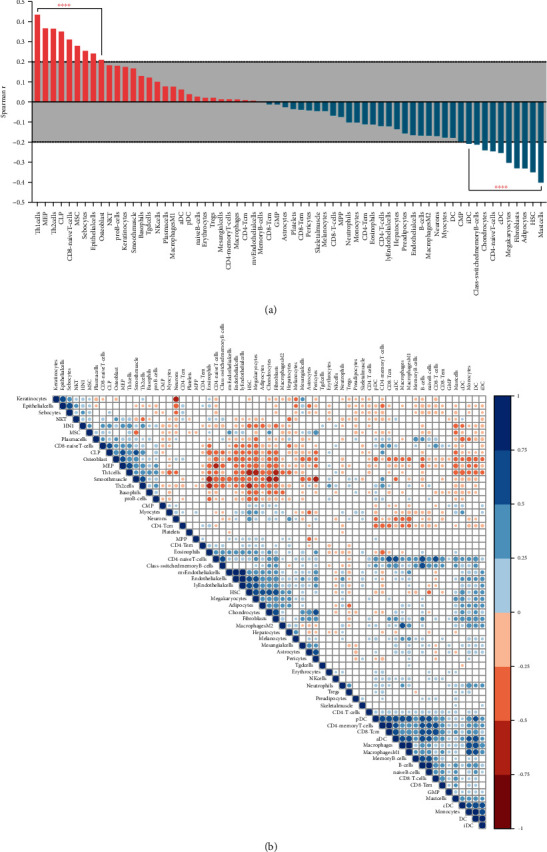
Correlation between HN1 and the abundance of microenvironment cells from xCell database. (a) Box plot of spearman *r* value from correlation analysis between HN1 and abundance of microenvironment cells. (b) Correlation heat map of HN1 and abundance of microenvironment cells.

**Figure 8:**
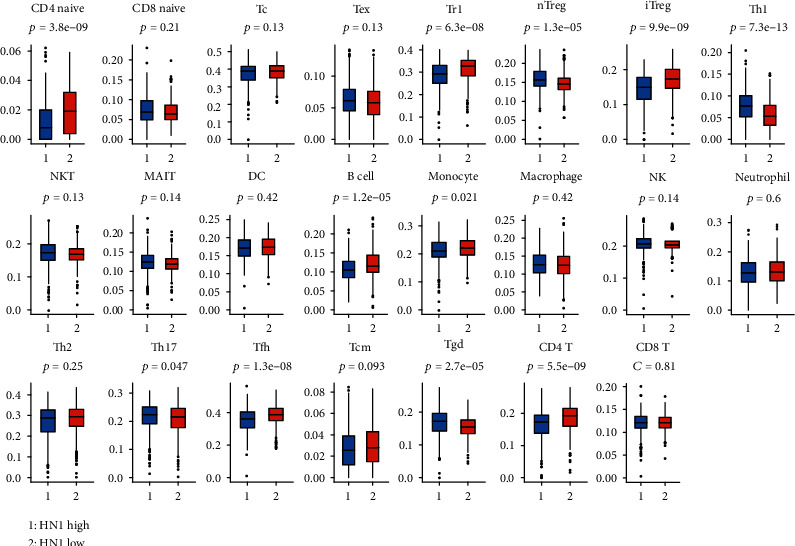
Comparison of the abundance of immune cells in HN1 high and HN1 low groups. The data are from ImmuCellAI database.

The correlation between HN1 and cancer microenvironment cells is presented in Figure 7 (Figure 7(a) is box plot of spearman *r* value; Figure 7(b) is correlation map). As we can see from Figure 7(a), HN1 is most correlated with Th1 cells, Th2 cells, CD8 naive T cells, etc., while negatively correlated with CD4 naive T cells, fibroblasts, mast cells, etc. Figure 7(b) is the correlation map of HN1 expression and abundance of stroma cells. It shows some intensity modules or clusters of different cell types.  Figure 8 is the box plot of cancer infiltrating immune cells in HN1 high and low groups. The abundance of CD4 T cells, iTreg cells, B cells, etc. were lower in HN1 high samples, while Th1 cells, nTreg cells, etc. were higher in HN1 high samples. Correlation map and box plot of spearman *r* are shown in Supplementary Figure 5. These results indicated that HN1 might play important roles in cancer progression by interacting with ICB-related factors and regulating biological behaviors of cancer microenvironment cells.

## 3. Discussion

Lung cancer is the leading cause of cancer death and the second most commonly diagnosed cancer, with an estimated 2.2 million new cancer cases and 1.8 million deaths worldwide in 2020 [6]. Big progresses have been achieved in clinical practices in recent years. KRAS and EGFR-targeted drugs were applied for lung cancer patients with specific mutations [7, 8]. Immune checkpoint blockade (ICB) therapies are also promising in the management of lung cancer [9]. However, therapeutic resistance emerges rapidly after treatment posing formidable obstacles to cancer therapeutics. Clarify the molecular mechanism lung cancer carcinogenesis and searching for novel prognostic biomarkers and therapeutic targets are of crucial importance.

THS is the residual of tobacco smoke that remains in the environments after active smoking. Several reports show that THS is a great public health hazard in indoor environment [10, 11]. For instance, THS could induce damage in human DNA and stimulate high levels of inflammatory cytokines and may involve in chronic obstructive pulmonary disease and asthma [5, 12]. Previously, Hang et al. reported that early exposure to THS was associated with increased lung cancer incidences in mouse model [4].

High-dimensional biological data resource is very important in screening new biomarkers and potential therapeutic targets [13]. In this study, the potential molecular mechanisms underlying the association between THS and LUAD were also explored using public available databases. Interestingly, we found THS-related DEGs were mainly associated the prognosis of patients with LUAD not LUSC. These results were in accordance with previous reports that THS was associated with increased lung adenocarcinoma incidences in mouse model.

Based on analysis results and literature mining, we choose HN1 for further investigation. HN1 was found to be overexpressed in LUAD and could serve as a prognostic biomarker for poor prognosis of patients with LUAD. Further analysis indicated that HN1 may promote LUAD progression by modulating tumor microenvironment and immune-related pathways. HN1, also named as JPT1, plays critical roles in the regulation of cell cycle and cell adhesion [14] and could negatively modulate AKT-mediated GSK3B signaling [15]. It has been reported that HN1 can promote tumorigenesis and metastasis in several cancer types such as breast cancer [16], prostate cancer [14], liver cancer [17], cervical cancer [18], and thyroid cancer [19]. However, this is the first report to show the association between HN1 and LUAD.

In summary, this is the first report using human data to show that THS-related transcriptional responses of respiratory epithelium are mainly associated with the development and progression of LUAD not LUSC. Exposure to THS is a significant health threat for nonsmokers, especially for children. The public should pay more attention to the potential risks of THS exposure, and potential buyers or renters of houses should be notified if there were THS risks in order to avoid unnecessary exposure to a potent lung carcinogen. Policy makers should also take THS into consideration when developing future environmental and health policies.

## 4. Materials and Methods

### 4.1. Ethics Statement

All the data were obtained from public datasets. The Research Ethics Committee of Zhejiang People's Hospital waived the requirement for ethical approval.

### 4.2. Data Sources

Gene expression data and protein data of LUAD and LUSC were downloaded from The Cancer Genome Atlas (TCGA: http://cancergenome.nih.gov/). THS-related gene expression data were obtained from Gene Expression Omnibus (GEO, accession no. GSE129959) [3, 20]. This dataset contains 4 participants that receive the clean air exposure first and THS exposure second. Abundance data of stroma and immune cells were obtained from xCell [21] and ImmuCellAI [22] databases.

### 4.3. Bioinformatics and Statistical Analyses

Survival analyses of 6 genes were analyzed online and downloaded from KmPlot [23] using a merged LUAD dataset. Differential expression and survival analyses of LUAD and LUSC TCGA data was performed using GEPIA version 2.0 [24]. UALCAN webtool was used for HN1 expression analysis in different stages of LUAD and normal control [25]. R 3.4.4 (R Foundation for statistical computing [http://www.r-project.org/]) was used for computing differentially expressed genes. Venn diagram analysis was performed using an online tool (http://bioinformatics.psb.ugent.be/webtools/Venn/). Pathway enrichment visualization was performed using NetworkAnalyst version 3.0 [26]. Gene set enrichment analysis (GSEA) was performed using clusterProfiler version 4.0 [27] and GSEA v4.0.3 (https://www.gsea-msigdb.org/gsea/downloads.jsp). Correlation analysis and visualization were performed using “corrplot” package. Other statistical analyses were performed using GraphPad Prism 5.01 (GraphPad Software, Inc. (http://www.graphpad.com)). Adjust *p* value was corrected for multiple comparisons using the Benjamini and Hochberg's false discovery rate [28]. Statistical significance was defined as a *p* value < 0.05.

## Figures and Tables

**Table 1:** Survival analyses of common DEGs in THS and lung cancer.

Gene symbol	LUAD		LUSC	
	Logrank *P*	Hazard ratio	Logrank *P*	Hazard ratio
C19orf48	6.60E-11	2.3 (1.78-2.97)	0.44	1.13 (0.83-1.54)
CENPM	1.30E-04	1.58 (1.25-1.99)	0.68	0.95 (0.75-1.21)
EXOSC5	1.20E-03	1.47 (1.16-1.85)	0.5	1.09 (0.86-1.37)
HIST2H4A	1.20E-05	1.68 (1.33-2.13)	0.7	0.95 (0.75-1.21)
HN1	1.00E-15	2.74 (2.12-3.54)	0.17	0.8 (0.59-1.1)
KRT17	5.70E-04	1.51 (1.19-1.9)	0.33	1.12 (0.89-1.42)
KRT8	1.60E-09	2.08 (1.63-2.66)	0.019	1.33 (1.05-1.68)
MUC20	1.60E-01	1.19 (0.93-1.51)	0.65	0.93 (0.68-1.27)
PAFAH1B3	0.0066	1.38 (1.09-1.75)	0.72	1.04 (0.82-1.32)
PPAP2C	6.30E-01	1.06 (0.84-1.33)	0.44	1.1 (0.87-1.39)
PSMG3	0.52	1.08 (0.85-1.38)	0.93	1.01 (0.74-1.38)
ROMO1	9.20E-06	1.77 (1.37-2.28)	0.49	1.12 (0.82-1.52)
GALE	0.0013	1.49 (1.17-1.9)	0.15	0.79 (0.58-1.09)
LGALS3BP	0.46	0.92 (0.73-1.15)	0.96	1.01 (0.79-1.27)
PDZK1IP1	0.0032	1.44 (1.13-1.84)	0.62	0.92 (0.68-1.26)
PRSS8	4.00E-01	1.11 (0.88-1.39)	0.45	0.91 (0.72-1.16)
TFF3	0.46	1.09 (0.87-1.38)	0.46	1.09 (0.86-1.39)
TMEM9	1.40E-06	0.55 (0.43-0.7)	0.87	1.03 (0.75-1.4)
TSTA3	8.40E-07	1.81 (1.43-2.3)	0.32	1.13 (0.89-1.43)
WFDC2	4.70E-01	1.09 (0.86-1.38)	0.98	1 (0.79-1.27)
ALDOA	2.50E-11	2.26 (1.77-2.89)	0.31	1.13 (0.89-1.43)
DPP3	1.60E-09	2.17 (1.68-2.81)	0.52	1.11 (0.81-1.51)
GSTP1	1.00E-04	1.61 (1.26-2.05)	0.31	1.13 (0.89-1.43)
MFAP2	3.90E-04	1.53 (1.21-1.94)	0.031	0.77 (0.61-0.98)
PHGDH	0.0059	1.38 (1.1-1.75)	0.2	1.17 (0.92-1.48)
POLD2	1.10E-05	1.7 (1.34-2.15)	0.24	1.15 (0.91-1.46)
RUVBL1	1.20E-06	1.8 (1.42-2.29)	0.9	0.99 (0.78-1.25)
SNRPB	2.30E-09	2.07 (1.62-2.64)	0.33	1.12 (0.89-1.42)
TUBG1	4.70E-05	1.63 (1.29-2.08)	0.33	0.89 (0.7-1.13)
HBG2	0.19	1.17 (0.93-1.47)	0.08	0.81 (0.64-1.03)
ADGRG6	0.14	0.83 (0.65-1.06)	0.49	1.12 (0.82-1.52)

## Data Availability

Data used in this study are all from publicly available datasets, and all data sources are noted in the manuscript.

## Supplementary Materials

Supplementary Materials Supplementary Figure 1: expression of HN1, KRT8, ROMO1, GSTP1, ALDOA, and SNRPB in other types of tumors in comparison to normal control. Red: high expression; blue: low expression. Supplementary Figure 2: prognostic value of HN1, KRT8, ROMO1, GSTP1, ALDOA, and SNRPB in other types of tumors. Red: good prognosis; blue: poor prognosis. Supplementary Figure 3: HN1 expression in different stages of LUAD is significantly higher in comparison to normal control. Supplementary Figure 4: high expression of HN1 is associated with poor prognosis of patients with LUAD. Supplementary Figure 5: correlation between HN1 and the abundance of immune cells from ImmuCellAI database. (A) Box plot of spearman *r* value from correlation analysis between HN1 and abundance of immune cells. (B) Correlation heat map of HN1 and abundance of immune cells. Supplementary Table 1: GSEA pathway enrichment of HN1-correlated genes.
