# Supplementary material for: Thirdhand Smoke May Promote Lung Adenocarcinoma Development through HN1
Source: Comput Math Methods Med. 2023 Jan 30;2023:3407313. doi: 10.1155/2023/3407313 (PMC9902119; doi:10.1155/2023/3407313)
Supplement: Supplementary Materials — Supplementary Figure 1: expression of HN1, KRT8, ROMO1, GSTP1, ALDOA, and SNRPB in other types of tumors in comparison to normal control. Red: high expression; blue: low expression. Supplementary Figure 2: prognostic value of HN1, KRT8, ROMO1, GSTP1, ALDOA, and SNRPB in other types of tumors. Red: good prognosis; blue: poor prognosis. Supplementary Figure 3: HN1 expression in different stages of LUAD is significantly higher in comparison to normal control. Supplementary Figure 4: high expression of HN1 is associated with poor prognosis of patients with LUAD. Supplementary Figure 5: correlation between HN1 and the abundance of immune cells from ImmuCellAI database. (A) Box plot of spearman r value from correlation analysis between HN1 and abundance of immune cells. (B) Correlation heat map of HN1 and abundance of immune cells. Supplementary Table 1: GSEA pathway enrichment of HN1-correlated genes. [file 3407313.f1.docx]

**Supplementary Materials**

**Supplementary Figures**

Supplementary Figure 1. Expression of HN1, KRT8, ROMO1, GSTP1, ALDOA and SNRPB in other types of tumors in comparison to normal control. Red: high expression; blue: low expression.


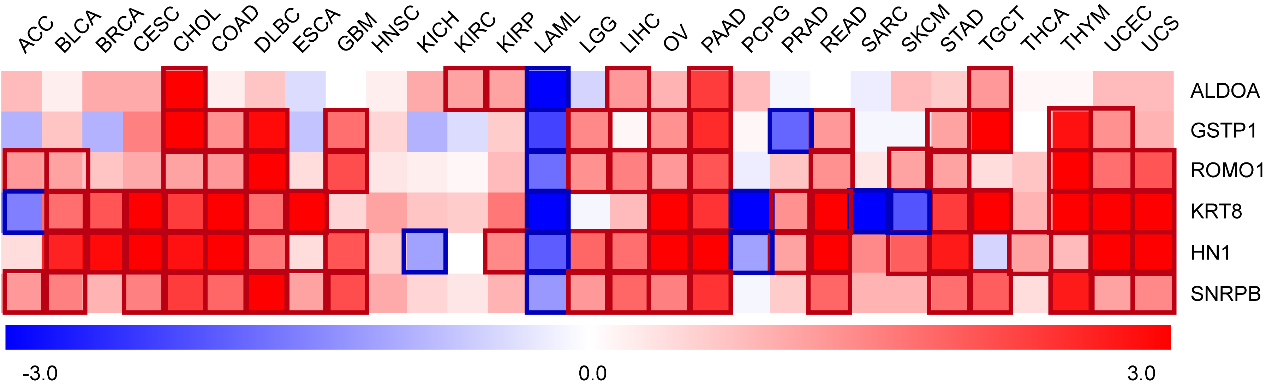


Supplementary Figure 2. Prognostic value of HN1, KRT8, ROMO1, GSTP1, ALDOA and SNRPB in other types of tumors. Red: good prognosis; blue: poor prognosis.


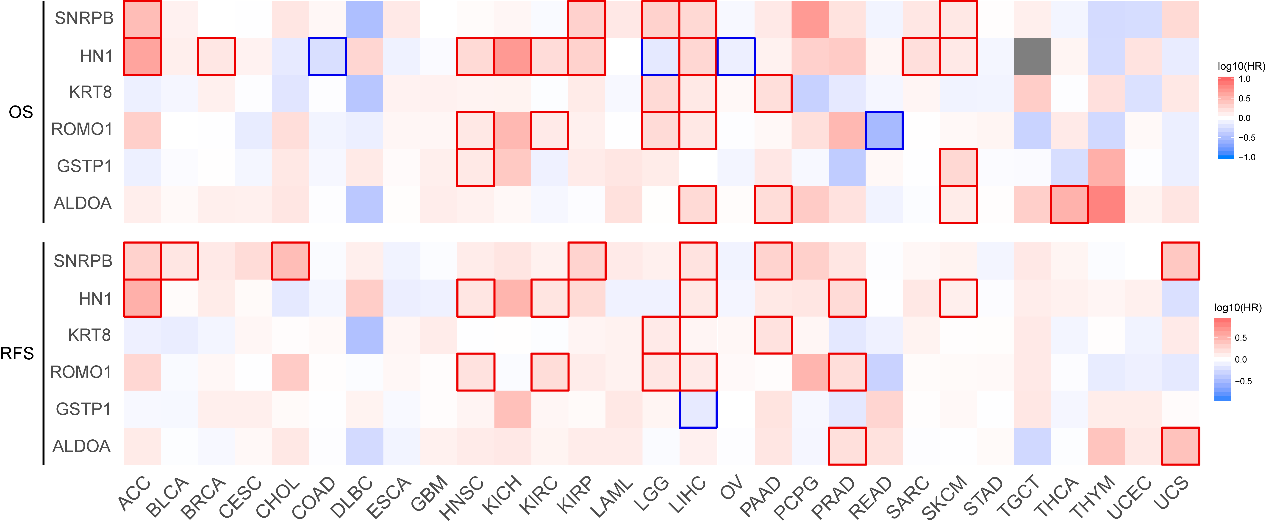


Supplementary Figure 3. HN1 expression in different stages of LUAD is significantly higher in comparison to normal control.


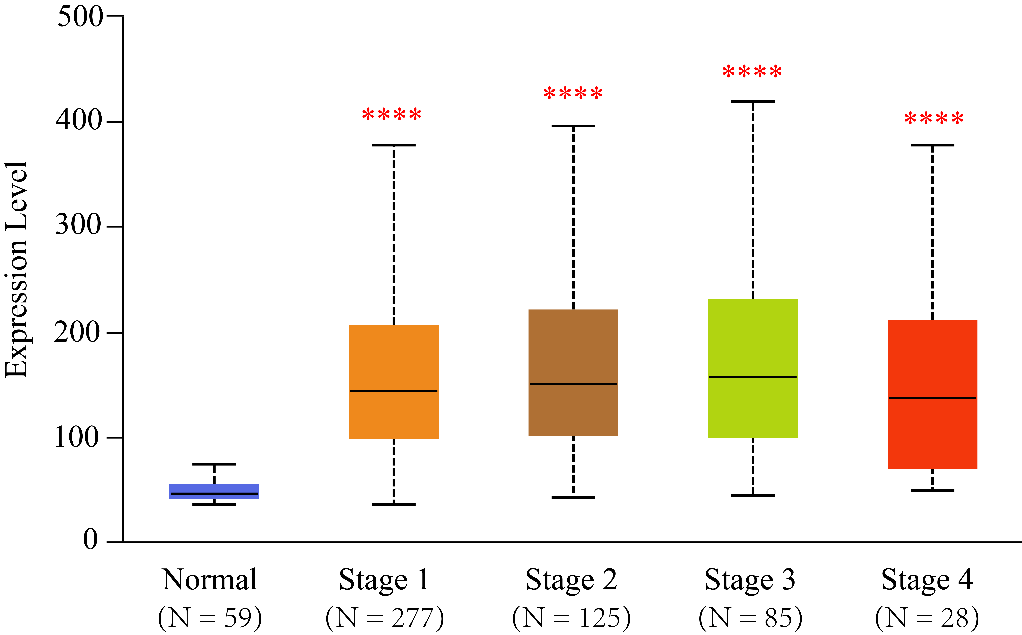


Supplementary Figure 4. High expression of HN1 is associated with poor prognosis of patients with LUAD.


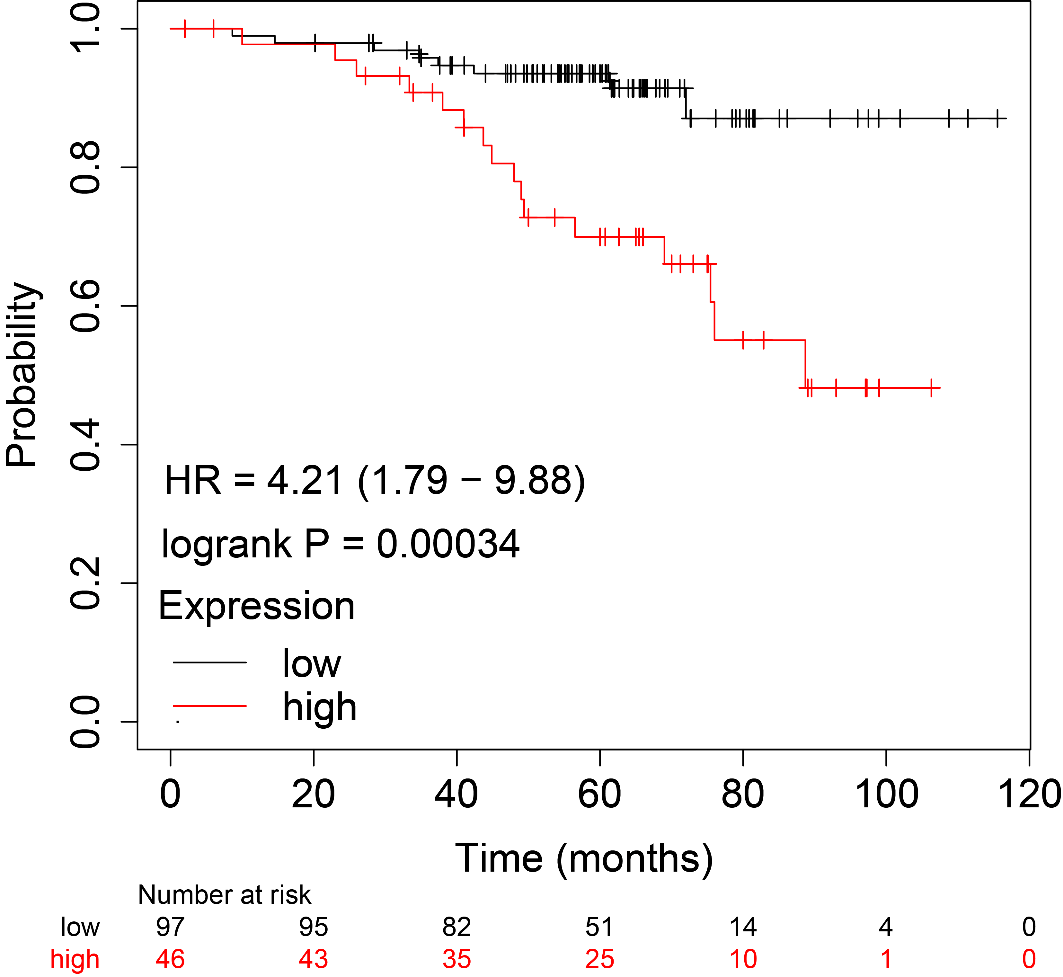


Supplementary Figure 5. Correlation between HN1 and the abundance of immune cells from ImmuCellAI database. (A) Box plot of spearman r value from correlation analysis between HN1 and abundance of immune cells. (B) correlation heat map of HN1 and abundance of immune cells.

**
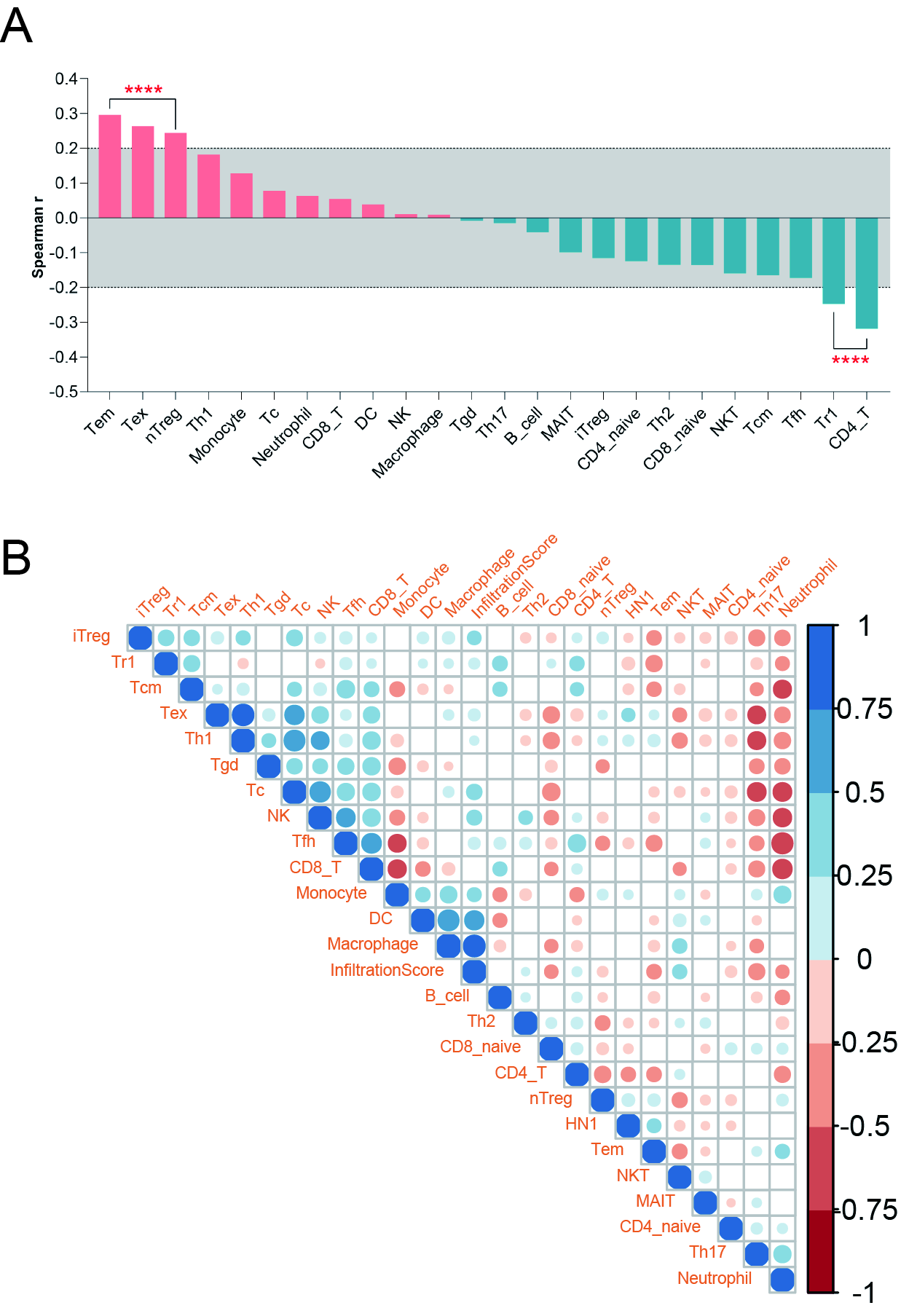
**

**Supplementary Tables**

Supplementary Table 1. GSEA pathway enrichment of HN1 correlated genes.

| Enriched KEGG pathway | Total | Hits | EnrichmentScore | P-value | P-adj value |
| --- | --- | --- | --- | --- | --- |
| cAMP signaling pathway | 30 | 17 | 0.444414338 | 0.000132767 | 0.001942316 |
| Calcium signaling pathway | 30 | 25 | 0.429959164 | 0.000134246 | 0.001942316 |
| cGMP-PKG signaling pathway | 34 | 22 | 0.436644556 | 0.000136874 | 0.001942316 |
| Cell adhesion molecules (CAMs) | 33 | 30 | 0.482714364 | 0.000137457 | 0.001942316 |
| ECM-receptor interaction | 133 | 96 | 0.474321348 | 0.000146606 | 0.001942316 |
| Hedgehog signaling pathway | 47 | 39 | 0.621385226 | 0.000161394 | 0.001942316 |
| Mismatch repair | 59 | 43 | -0.802742758 | 0.000248447 | 0.001942316 |
| DNA replication | 36 | 33 | -0.785093506 | 0.000262123 | 0.001942316 |
| Proteoglycans in cancer | 56 | 51 | 0.374704399 | 0.000264971 | 0.001942316 |
| Platelet activation | 31 | 28 | 0.421674366 | 0.000285592 | 0.001942316 |
| Inflammatory mediator regulation of TRP channels | 48 | 42 | 0.449458025 | 0.000292013 | 0.001942316 |
| p53 signaling pathway | 20 | 18 | -0.442843162 | 0.000295247 | 0.001942316 |
| Oxidative phosphorylation | 29 | 24 | -0.653800104 | 0.000324254 | 0.001942316 |
| Cell cycle | 27 | 27 | -0.654106636 | 0.000331675 | 0.001942316 |
| Cellular senescence | 28 | 22 | -0.359462489 | 0.000359195 | 0.001942316 |
| PI3K-Akt signaling pathway | 20 | 18 | 0.314168518 | 0.000372949 | 0.00195006 |
| Viral carcinogenesis | 30 | 25 | -0.358007106 | 0.000377644 | 0.00195006 |
| Focal adhesion | 26 | 24 | 0.366785786 | 0.00039968 | 0.002012183 |
| Wnt signaling pathway | 67 | 50 | 0.410081027 | 0.000416782 | 0.00206272 |
| Rap1 signaling pathway | 66 | 27 | 0.358975883 | 0.000530223 | 0.002497178 |
| MAPK signaling pathway | 79 | 50 | 0.312115254 | 0.001654997 | 0.006117203 |
| Th1 and Th2 cell differentiation | 212 | 173 | 0.395968035 | 0.005845324 | 0.017596232 |
| Jak-STAT signaling pathway | 294 | 243 | 0.355291803 | 0.006137596 | 0.018287532 |
| Drug metabolism - cytochrome P450 | 124 | 103 | 0.408001855 | 0.010359537 | 0.028270886 |
| EGFR tyrosine kinase inhibitor resistance | 123 | 104 | 0.387593448 | 0.016343826 | 0.041499105 |
| PPAR signaling pathway | 153 | 113 | 0.386357395 | 0.019408643 | 0.047624569 |
